# Retinal self-organization: a model of retinal ganglion cells and starburst amacrine cells mosaic formation

**DOI:** 10.1098/rsob.220217

**Published:** 2023-04-05

**Authors:** Jean de Montigny, Evelyne Sernagor, Roman Bauer

**Affiliations:** ^1^ Biosciences Institute, Newcastle University, Newcastle upon Tyne NE1 7RU, UK; ^2^ Department of Computer Science, University of Surrey, Guildford GU2 7XH, UK

**Keywords:** self-organization, retinal mosaic, computational modelling, retinal ganglion cells, starburst amacrine cells

## Abstract

Individual retinal cell types exhibit semi-regular spatial patterns called retinal mosaics. Retinal ganglion cells (RGCs) and starburst amacrine cells (SACs) are known to exhibit such layouts. Mechanisms responsible for the formation of mosaics are not well understood but follow three main principles: (i) homotypic cells prevent nearby cells from adopting the same type, (ii) cell tangential migration and (iii) cell death. Alongside experiments in mouse, we use BioDynaMo, an agent-based simulation framework, to build a detailed and mechanistic model of mosaic formation. We investigate the implications of the three theories for RGC's mosaic formation. We report that the cell migration mechanism yields the most regular mosaics. In addition, we propose that low-density RGC type mosaics exhibit on average low regularities, and thus we question the relevance of regular spacing as a criterion for a group of RGCs to form a RGC type. We investigate SAC mosaics formation and interactions between the ganglion cell layer (GCL) and inner nuclear layer (INL) populations. We propose that homotypic interactions between the GCL and INL populations during mosaics creation are required to reproduce the observed SAC mosaics' characteristics. This suggests that the GCL and INL populations of SACs might not be independent during retinal development.

## Graphical abstract

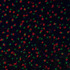

## 1. Introduction

**Figure 1.**
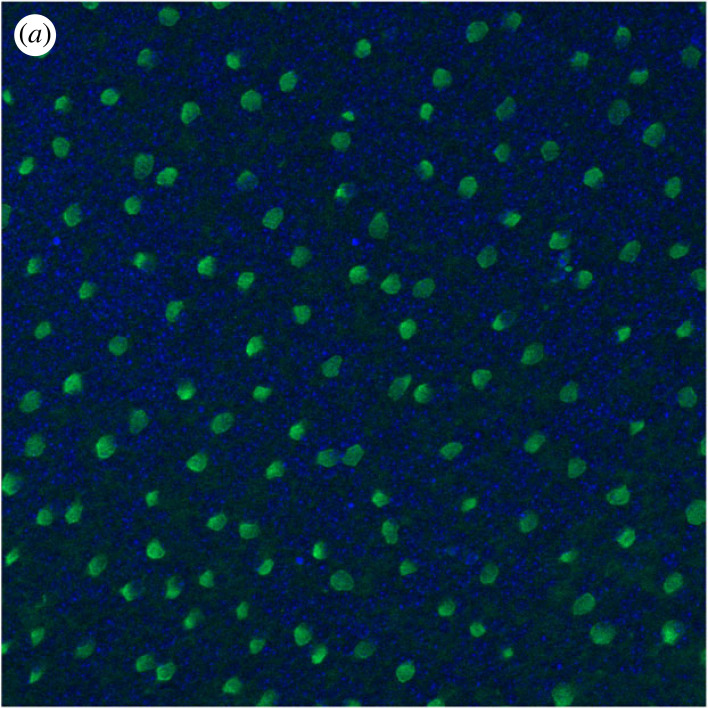
SAC mosaic in the INL level, obtained by a ChAT immunostaining in a P9 pup retina.

The mammalian retina, including mouse (as used in this work), is composed of six main types of neuronal cells, namely cones, rods, horizontal, bipolar, amacrine and ganglion cells. These can be sub-divided into many different anatomical and functional subtypes, forming a complexly organized structure. Notably, individual cell types exhibit semi-regular spatial patterns called mosaics. Regular spacing between homotypic cells enable homogeneous processing of the light signals, leaving no perceptual holes within our visual field. Sub-groups of retinal ganglion cells (RGCs) and starburst amacrine cells (SACs) are known to form regular mosaics (figure 1), and both cell types are widely used to study mosaic organization. SACs are divided into two populations, located in the inner nuclear layer (INL) and in the ganglion cell layer (GCL). Each population forms a mosaic [1,2].

RGCs, located in the GCL, are the output cells of the retina, sending all visual information processed in the retina to the visual areas of the brain. There are many ways RGCs can be classified into sub-groups. One approach is to classify them into three functional and morphological groups depending on the sub-layer their dendrites laminate into in the inner plexiform layer, forming the On, Off and On-Off groups. In mouse, RGCs can however be divided into more than 40 types [3–6], each having different functional and anatomical characteristics. The number of RGCs sub-group varies between species, and for a different species, the density of these sub-groups is also known to greatly differ, varying from less than 50 cells mm^−2^ to more than 300 cells mm^−2^ [5]. It has been proposed that a group of RGCs has to fulfil four criteria in order to be considered a RGC type [5]: (i) morphological homogeneity (dendritic tree shape); (ii) identical physiological properties (electrophysiological response to light); (iii) similar gene expression (molecular signature); and (iv) regular spacing (mosaic). Thus, being organized in mosaics could represent an important feature of each RGC type. Even if the total number of RGC types is estimated to more than 40, only 19 have been fully characterized (cellular density, morphology, molecular signature and functions) [5]. Other RGC types have been only partially characterized. While RGCs dendritic arbours are also known to form mosaics through homotypic self-avoidance [4,7,8], our work will only focus on cell bodies.

RGCs are the only cell class notably more numerous in the immature retina than in the adult retina. Indeed, around 60% of newly born RGCs undergo programmed cell death (CD) (apoptosis) during the perinatal period [9]. Much remains to be discovered about the impact of RGC apoptosis on the maturation of retinal circuitry and visual pathways, even if studies already suggest that apoptosis could be implicated in the development of some RGCs connectivity [10].

Despite being an important feature of retinal organization, retinal mosaic’ formation is not fully understood yet. In particular, three mechanisms are believed to potentially take part in their development: cell fate (CF) determination, programmed CD and tangential cellular migration (CM) [4,11].

### 1.1. Cell fate determination

CF is a process by which a cell of a certain type will prevent the emergence of same type cells in its vicinity [12]. After passing through an intrinsically determined state, retinal progenitors are left in an undifferentiated state, but are now only capable of giving rise to a limited subset of cell types. The precise type the cells choose to differentiate into depends on extrinsic signals [13]. These extrinsic signals can consist of chemical cues such as trans-membrane proteins [14–16] and may be delivered by an already differentiated retinal cell in order to block neighbouring undifferentiated cells from differentiating into the same cell type. Such a process has been previously demonstrated to be implicated in the tiling of the Drosophila photoreceptor R8, which prevents neighbouring cells to differentiate into the R8 type [17]. Recent studies in mouse also show evidences of a signalling cascade influencing the developmental pathway of RGC type specification, through a competitive mechanism requiring local signalling [18] and that RGCs types are not defined after mitosis, but acquired through fate restriction [19].

### 1.2. Programmed cell death

RGCs exhibit a very high rate of programmed death (60–70% of the initial population [20]) during normal development. The CD mechanism is believed to be implicated in the selection of relevant cells in order to build a functional retina. Following this principle, cellular death has been proposed to be a consequence of RGCs not being able to establish correct axonal connections in the lateral geniculate nucleus in the thalamus [21]. RGC CD has also been shown to depend on neighbouring cells' electrical activity [22,23]. Creating either spatial or functional competition between homotypic cells could lead to the formation or refinement of mosaics. Due to major differences in death rate, the importance of programmed CD upon mosaic formation seems however to vary between cell classes, and even between sub-groups within the same cell class. CD has been proposed to contribute to mosaic formation for several cell groups in the retina, including amacrine cells [8,24] and at least one RGC type [23].

### 1.3. Cellular migration

All retinal cells undergo migration during retinal development, both vertical (from one layer to another) and tangential (within the same layer). Cells can move between 20 µm and 100 µm tangentially from their initial location [25,26]. This has been demonstrated for SACs mosaic formation, where homotypic cells move tangentially away from each other [27]. CM is believed to be key mechanism in mosaic formation [8]. Mechanisms responsible for this migration are not fully understood, even if chemical cues seem to play a key role, such as in the case of SACs [1]. Diffusible signals or contact-mediated interactions between homotypic cells may be responsible for mosaic formation [28]. Dendritic contact-mediated interactions have been shown to play a role in tangential migration [1,29,30]. However, these dendritic interactions may not be necessary for all mosaic formation as mosaics appear, partially or completely, before extensive dendritic growth [28,31] and thus without contact-mediated interactions. Thus, cell–cell interactions seem to play an important role for tangential migration.

Of course, it is likely that the formation of mosaic patterns is due to the combinations of all three mechanisms [31]. Previous mathematical simulations of retinal mosaic formation have been conducted [11,22]. These studies investigate the involvement of the CF, CD and CM mechanisms, suggesting a central role for the CM mechanism. They show that regular mosaics can arise solely from interactions between cells, using CD and CM mechanisms, and that the latter yields the most regular mosaics. They also show that CF is the least effective mechanism to create regular mosaics but improves the effect of CD if CF and CD are modelled sequentially. However, these studies are highly abstract and do not mechanistically model retinal mosaic formation, thus limiting their biological interpretation. No mechanistic model of mosaic formation currently exists.

Agent-based (AB) modelling is a type of computational modelling in which each simulation object is an autonomous agent. Despite the absence of any global supervisor, highly complex structures can emerge from local interactions of agents that self-organize [32,33]. This approach is particularly relevant to model biological phenomena where cells exhibit this characteristic as well and allows the construction of mechanistic and realistic models of retinal mosaic formation.

The impact and implications of all mechanisms involved in mosaic formation (CF, CD and CM) are not fully understood, and much remains to be done in order to establish the detailed mechanisms governing mosaic formation. In this work, we analyse mechanisms underlying retinal mosaics self-organization using AB computational modelling. In particular, the biological requirements and the effect of individual mechanisms generating these cellular patterns are investigated. We also acquired experimental data to inform our computational modelling and validate the results of our simulations.

## 2. Methods

### 2.1. Experimental work

#### 2.1.1. Immunohistochemistry

Retinal wholemounts were prepared from mouse pups aged P2–P11, flattened on nitrocellulose membrane filters and fixed for 45 min in 4% paraformaldehyde. Retinas were then incubated in blocking solution—5% of secondary antibody host species serum with 0.5% Triton X-100 in 0.1 M phosphate buffer solution (PBS)—for 1 h.

Retinas were incubated with 0.5% Triton X-100 with RBPMS (1 : 500) and ChAT (1 : 500) in PBS for 3 days at 4°C, then washed with PBS and incubated with 0.5% Triton X-100 with donkey anti-rabbit Alexa 568 (1 : 500) and donkey anti-goat Dylight 488 (1 : 500) in PBS for 1 day at 4°C. Finally, retinas were washed with PBS and embedded with OptiClear. Primary antibodies used were ChAT (AB144P, goat polyclonal, Merck Millipore) for SACs staining and RBPMS (1830-RBPMS, rabbit polyclonal, Phosphosolutions) for RGCs staining. Secondary antibodies used were Donkey anti-rabbit Alexa 568 (A10042, Invitrogen) and Donkey anti-goat Dylight 488 (SA5-10086, ThermoFisher Scientific).

Zeiss AxioImager with Apotome processing and Zeiss LSM 800 confocal microscope were used to image the retinas. Stitching of adjacent areas was achieved to image the whole retinal surface at high-resolution. Images at 40× magnification were acquired in mid-peripheral regions to perform cell count and mosaic regularity measures.

#### 2.1.2. Cell populations density

The average RGC and SAC density for each developmental day was measured by performing a manual cell count from P2 to P10 for RGCs and from P4 to P10 for SACs. By accounting for the surface expansion observed during retinal development, we estimated changes in populations through development. The estimated total RGC and SAC populations for a given retina are calculated by multiplying the averaged cell density (obtained from 3 to 6 sample areas per retina) by its corresponding retinal surface. These individual measurements are then averaged for each developmental day to give an estimation of the total population from P2 to P10. Cell population death rate during development is then calculated.

#### 2.1.3. Starburst amacrine cell mosaics

Positions of SACs in the GCL and INL are extracted in order to calculate mosaic regularities of these two populations from P4 to P10. A measure of GCL and INL mosaics exclusion has also been conducted. The calculated exclusion factor is based, for two distinct populations, on a count of cells from the first populations located within a determined distance (exclusion diameter) from cells belonging of the second population. This score is then normalized, to give an exclusion factor between 0 and 1. 1 denotes a perfect exclusion, meaning that all cells of the first population are located at a distance greater than the exclusion diameter from all cells of the second population. By consequence, only exclusion factors calculated with an identical exclusion diameter can be compared. A unique exclusion diameter of 32 µm has been chosen here, corresponding to about three times the diameter of a SAC soma, and allowing a good discrimination between our different mosaics.

### 2.2. Biodynamo

Simulations were conducted using the AB simulation framework BioDynaMo [34].

Each simulation object in BioDynaMo possesses its own characteristics, such as its three-dimensional geometry, mass and position in space. Individual neurons are represented by a sphere. Diffusion in three-dimensional of chemical substances in the extracellular space is implemented, with the discrete central difference method. This diffusion is supported by grids representing substances concentration and gradients. Mechanical forces are also considered between all simulation objects such that they cannot overlap, but mechanically repulse each other. Each simulation object can have a *biology module* attached to it that describes its behaviour at each simulation time step, such as substance secretion, CM or cell growth.

As an AB simulation framework, each simulation object is independent, without a central organization unit that orchestrates the behaviour of all simulation objects. Thus, simulation objects only have access to their micro-environment, which consists of other objects and chemical substances of the extracellular matrix in their proximity.

Several biology modules have been defined and used in our simulations, in order to describe cells behaviour for self-organization (CF, CD and CM), cell growth and chemical substances secretion.

### 2.3. Simulations

Cells of 7 to 8 µm diameter are randomly distributed (uniform distribution) in a space of 1000 m × 1000 µm × 22 µm, creating a multi-layer plane. Simulation space borders have been set to 1300 µm to avoid side effect from cells located to the edge. If CD is simulated, the initial cell density is set to 8600 cells mm^−2^, in order to reach the RGC density once the CD mechanism is over—around 3000 cells mm^−2^ reported in literature [5] and around 3500 cells mm^−2^ in our measures. If CD is not simulated, the initial cell density is set to 3000 cells mm^−2^. No additional cells are created during the simulation. As cell density decreases due to CD (and as cell diameter increases up to 14 µm), the initial multi-layer collapses into a RGC monolayer, accordingly to our *in vitro* observation. This collapse is not implemented depending on physical pressure as it is the case *in vivo*, but by having cells moving along the *z*-axis toward the centre of the RGC layer. Time step is set such that 160 steps simulate 1 day of development. Mosaic formation simulations run for a maximum of 2240 steps, corresponding to 14 days of development.

In our simulations, the global RGC population is sub-divided into 43 types. Some have been precisely documented, such as the On or On-Off direction-selective RGCs or the Off J-RGCs, and their population densities and dendritic arbours characteristics are known. However, these precisely documented RGC groups represent only 19 types and merely about 60% of the total RGC population (approx. 1700 cells mm^−2^ over approximately 3000 cells mm^−2^) [5]. RGC types contributing to the remaining 40% of the population have been estimated using results from Sanes & Masland [5], Reese & Keeley [4] and Baden *et al*. [3]. These authors state that numerous RGC types are still unknown, and these cells are probably sparsely distributed across the retina. Thus, in addition of the 19 precisely documented RGC types, we implemented 24 RGC types of various but low densities, for a total of 43 RGC types. This final number is also in accordance with previous studies classifying RGCs into 42 types based on visually evoked responses [35]. All implemented RGC types and their corresponding starting and final densities are summarized in table 1.

**Table 1.** Implemented RGC types and parameters used for different conditions. D: death mechanism only. FD: fate and death mechanisms. FDM: fate, death and migration mechanisms. death: concentration threshold for death mechanism. Migration: concentration threshold for migration mechanism.

cell type	type name	start density cells mm^−2^	final density cells mm^−2^	D	FD	death	FDM
0	on-off_dsgca	357	125	2.0367	2.0334	2.023	2.02
1	on-off_dsgcb	357	125	2.0367	2.0334	2.023	2.02
2	on-off_dsgcc	357	125	2.0367	2.0334	2.023	2.02
3	on-off_dsgcd	357	125	2.0367	2.0334	2.023	2.02
4	on-off_m3	57	20	1.9872	1.9855	1.9855	1.983
5	on-off_led	714	250	2.116	2.098	2.08	2.065
6	on-off_u	57	20	1.9872	1.9855	1.985	1.983
7	on-off_v	57	20	1.9872	1.9855	1.985	1.983
8	on-off_w	171	60	2.001	1.9978	1.996	1.994
9	on-off_x	143	50	1.9968	1.9945	1.994	1.9925
10	on-off_y	114	40	1.994	1.993	1.993	1.991
11	on-off_z	114	40	1.994	1.993	1.993	1.991
100	on_dsgca	114	40	1.994	1.993	1.993	1.991
101	on_dsgcb	114	40	1.994	1.993	1.993	1.991
102	on_dsgcc	114	40	1.994	1.993	1.993	1.991
103	on_aplha	114	40	1.994	1.993	1.993	1.991
104	on_m2	160	56	2	1.9953	1.994	1.992
105	on_m4	57	20	1.9872	1.9855	1.985	1.983
106	on_m5	57	20	1.9872	1.9855	1.985	1.983
107	on_o	428	150	2.05	2.0425	2.035	2.031
108	on_p	286	100	2.022	2.018	2.012	2.01
109	on_q	286	100	2.022	2.018	2.012	2.01
110	on_r	228	80	2.011	2.0082	2.004	2.002
111	on_s	171	60	2.001	1.9978	1.995	1.993
112	on_t	171	60	2.001	1.9978	1.995	1.993
113	on_u	143	50	1.9968	1.9945	1.994	1.9925
114	on_v	143	50	1.9968	1.9945	1.994	1.9925
115	on_w	97	34	1.993	1.9934	1.989	1.987
116	on_x	57	20	1.9872	1.9855	1.985	1.983
117	on_y	57	20	1.9872	1.9855	1.985	1.983
118	on_z	57	20	1.9872	1.9855	1.985	1.983
200	off_aplhaa	114	40	1.994	1.993	1.993	1.991
201	off_aplhab	114	40	1.994	1.993	1.993	1.991
202	off_m1	180	63	2.006	1.9979	1.998	1.996
203	off_j	571	200	2.078	2.065	2.058	2.049
204	off_mini_j	1000	350	2.179	2.155	2.134	2.098
205	off_midi_j	228	80	2.011	2.0082	2.004	2.002
206	off_u	57	20	1.9872	1.9855	1.985	1.983
207	off_v	57	20	1.9872	1.9855	1.985	1.983
208	off_w	171	60	2.001	1.9978	1.995	1.993
209	off_x	143	50	1.9968	1.9945	1.994	1.9925
210	off_y	114	40	1.994	1.993	1.993	1.991
211	off_z	106	37	1.9935	1.9928	1.989	1.988

Cells are created with no predefined types when simulating the CF mechanism. Otherwise, cells are created with a type matching the theoretical initial density of each RGC type.

#### 2.3.1. Substance secretion

Each RGC type secretes a specific chemical substance that diffuses in the extracellular space, using grids of 2 µm^3^ voxels. At each time step, the concentration value of each voxel is updated according to the equation

$$\begin{array}{c} u_{i,j,k}^{n}+1=(u_{i,j,k}^{n}+\frac{\nu \Delta t}{\Delta x^{2}}(u_{i+1,j,k}^{n}-2u_{i,j,k}^{n}+u_{i-1,j,k}^{n})\;\;\\ +\frac{\nu \Delta t}{\Delta y^{2}}(u_{i,j+1,k}^{n}-2u_{i,j,k}^{n}+u_{i,j-1,k}^{n})+\frac{\nu \Delta t}{\Delta z^{2}}(u_{i,j,k+1}^{n}-2u_{i,j,k}^{n}+u_{i,j,k-1}^{n}))\times (1-\mu ),\; \end{array}$$

where $u_{i,j,k}^{n}+1$ is the concentration value on grid point (*i*, *j*, *k*) at time step *n* + 1, *ν* is the diffusion coefficient (2 in our simulations), *μ* is the decay constant (0 in our simulations), Δ*t* is the duration of one time step, and Δ*x*, Δ*y*, Δ*z* are the distances between grid points in the *x*, *y*, *z* directions, respectively. The secretion corresponds to an increase of substance concentration by 1 at the cell centre position at each time step. Each cell uses only its cell type chemical substance concentration as a developmental cue for the three mosaic formation mechanisms. Undifferentiated cells do not secrete any substance. Simulations are initialized without any substance pre-existing in the extracellular space.

#### 2.3.2. Retinal ganglion cell mosaic formation: cell fate

CF is implemented such that substances act as an inhibitor for cell differentiation, preventing nearby undifferentiated cells to adopt the same types. RGC cells can choose to differentiate only into non-inhibited RGC types. If multiple types are not inhibited, the selected type depends on a probability. When implemented, CF is the first event to occur during simulations and no other mechanism occurs concomitantly. CF mechanism is over when all undifferentiated cells from the initial pool have selected to become a specific cell type.

#### 2.3.3. Retinal ganglion cell mosaic formation: cell death

The CD mechanism corresponds to the cells removing themselves from the simulation if their corresponding substance concentration is higher than a defined threshold (table 1). Thereby, the clusters of homotypic cells exhibit high death rates and become sparser. If CF is implemented, CD is triggered after completion of CF and continues until a steady state is reached. This steady state is reached by P5–P6 and is achieved without global controllers but depends on the chosen concentration threshold triggering CD.

#### 2.3.4. Retinal ganglion cell mosaic formation: cell migration

CM is implemented such that the homotypic substances act as a repulsive factor. Thereby, cells exhibit short-distance avoidance, moving tangentially against their substance gradient, distancing themselves from homotypic neighbours. CM chemical concentration threshold is always set at a lower value than the CD threshold (table 1) such that CM is triggered before CD. We assume that CM is triggered after completion of CF, at the same time as CD, and continues either until a steady state or day 13 is reached.

Development conditions incorporating all combinations of these three mechanisms have been investigated.

**Figure 2.**
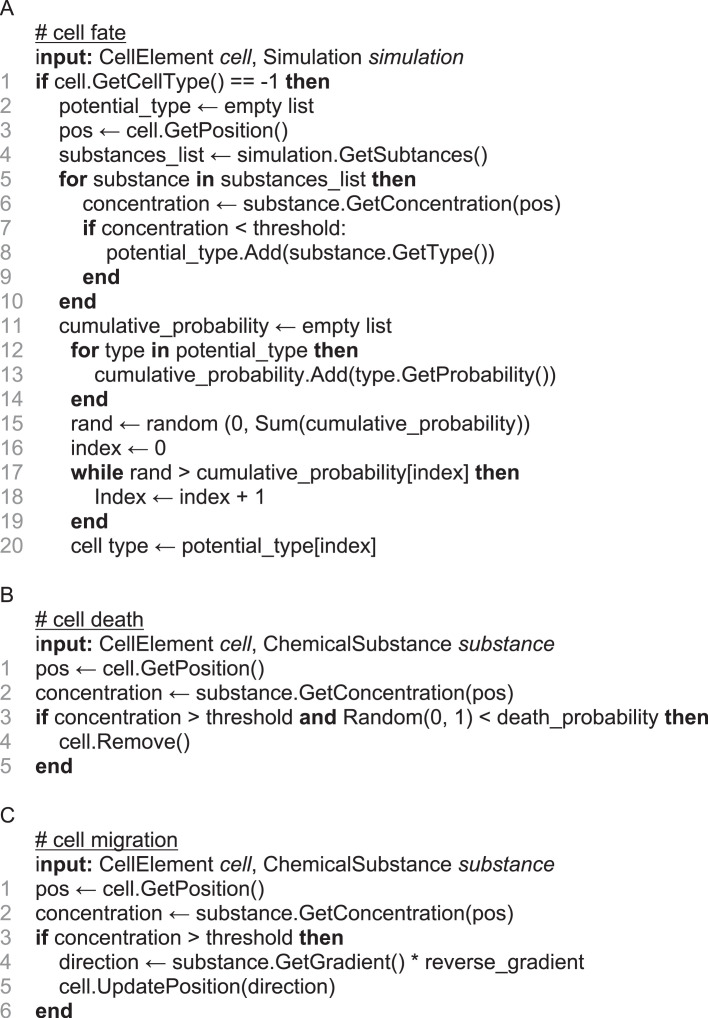
Pseudocode describing biology modules. **A:** CF. **B:** CD. **C:** CM.

Pseudocode corresponding to these three *biology modules* can be found in (figure 2), and schematic representations can be found in electronic supplementary material, figure S1.

#### 2.3.5. Parameter tuning

To model realistic mechanisms of CD and CM, parameters have been set to match experimental measures in the retina. The CD parameter corresponds to the chemical cue concentration threshold triggering the CD mechanism. For each population, this threshold parameter is set such that the death rate steady state matches the measured CD *in vitro*—between 60% and 65% of death rate [9,20]. For a specific population, the CD parameter differs between simulations implementing CD alone or in the case of a combination of CD and CM mechanisms. Indeed, by migrating cells away from the most concentrated chemical cue region, CM decreases the death rate. A lower CD threshold must be used in order to replicate the observed death rate of 60–65%. Likewise, if CF and CD are simulated together this parameter differs, as CF increases the regular spacing of a cell population compared to random distribution and so close homotypic cells are less common.

The CM parameter corresponds to the chemical cue concentration triggering the CM mechanism. This threshold parameter is set depending on the CD parameter, always being lower such that CM is triggered before CD. This parameter is also set such that interaction distance between cells is restricted to local interactions and such that CM distance does not exceed *in vivo* measures (average migration distance experimentally measured at around 20 µm [27], and not exceeding 30 µm [31]).

As the mechanisms influence each other, parameters vary depending on the implemented mechanisms. Table 1 summarizes the parameters used for RGC mosaics formation mechanisms.

#### 2.3.6. Starburst amacrine cell mosaic formation

The simulation of SAC mosaic formation is achieved using the CM mechanism. Once mosaics are formed, the two populations (GLC and INL populations) migrate to their respective layers, in the GCL and INL. This migration is achieved by migrating cells along the *z*-axis depending on their cell type (GCL or INL population). CM parameter has been set such that the mosaics Regularity Index (RIs) match the measured RIs in mouse SACs mosaics. Importantly, CM concentration thresholds are identical for the GCL and INL populations.

### 2.4. Data analysis

The RI was used to assess the regularity of the mosaics. It is computed as the average value of the closest neighbour distribution (distribution of the closest neighbour measured for each cell) divided by its s.d. [36]. The RI offers a single score that can discriminate regularity differences between mosaics of low regularities. In addition, and as previously reported [4], the RI offers a scale-invariant measure of mosaic regularity and thus more direct evidence of any change in the mosaic spatial organization during development. It is not only the absolute RI value that carries information, but also its evolution across development, related to the contribution of each mosaic developmental mechanism (CF, CD and CM). However, RI is sensitive to a low sampling rate, leading to significant variability in RI scores for mosaics constituted of few cells. The RI of a random distribution is between 1.8 and 2.

Comparisons between two RI values have been conducted using Mann–Whitney *U*-test as Kolmogorov–Smirnov tests have revealed non-normal distribution of RI values.

## 3. Results

### 3.1. Retinal ganglion cell mosaic development

**Figure 3.**
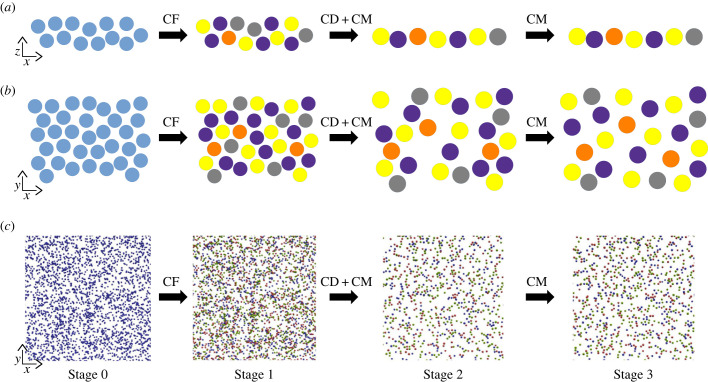
Time course and spatial structure of a simulation using CF, CD and CM mechanisms. (*a,b*) schematic course of a simulation in *x*,*z* and *x*,*y* orientation, respectively. Undifferentiated cells are represented in blue and differentiated cells (RGC types) in yellow, indigo, grey and orange. For clarity purpose, only 4 out of the 43 implemented types are represented. (*c*) Time lapse of mosaic formation using BioDynaMo. Stage 0: First step of the simulation. Stage 1: Simulation after the end of CF mechanism, at day 1 (step 180). Average RI = 2.41 ± 0.1. Stage 2: Simulation after the end of CD mechanism, at day 6 (step 600). Average RI = 3.42 ± 0.31. Stage 3: Simulation at the end of the CM mechanism, at day 14 (step 2240). Average RI = 3.99 ± 0.36. Undifferentiated cells and represented in blue. On cells are represented in green, Off cells in red and On-Off cells in purple. The On, Off and On-Off groups are not RGC types but groups of types and thus do not form mosaics at the group level while each RGC type composing the group forms an individual mosaic. If CF mechanism is not simulated, simulations start at stage 1 with a defined cell type attributed at cells creation, accordingly to their type's theoretical density.

Different steps of a simulation implementing the CF, CD and CM mechanisms are illustrated by figure 3. Several simulation conditions have been investigated, implementing either a single mechanism (CF, CD or CM) or combinations of mechanisms (CF-CD and CF-CD-CM). As the CD and CM mechanisms require cells to be differentiated, CF is simulated beforehand. If CF is not simulated, cells are created with a defined type at their creation, accordingly to their type's theoretical density. All cells are differentiated at the end of simulation day 1, and CD mechanism has reached a steady state at the end of simulation day 6.

To achieve realistic simulations, parameters tuning is crucial. For this reason, parameters have been set to match experimental measures in the retina, as described in the Methods.

#### 3.1.1. Cell fate

**Figure 4.**
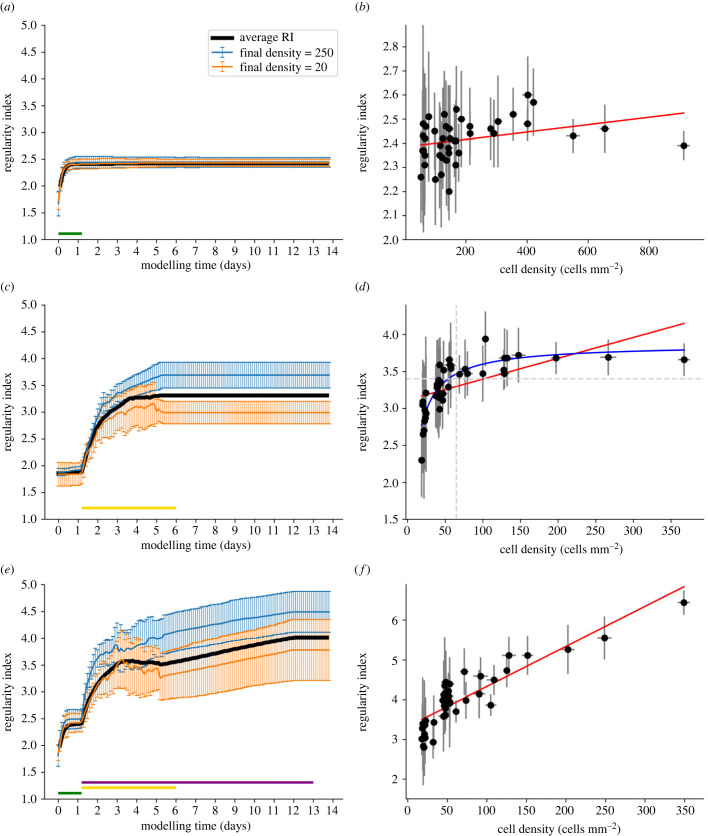
RGC mosaic formation modelling using an ABM approach. (*a,b*) CF mechanism only. (*c,d*) CD mechanism only. (*e,f*) Combination of CF, CD and CM combination. (*a,c,e*) RI score evolution during simulation (*x*-axis: 1 developmental day in mouse corresponds to 160 simulation steps). Average RI values for all RGC types are displayed in black while two populations of high and low densities (250 and 20 cells mm^−2^, respectively) are displayed in blue and orange. The green, yellow and purple horizontal lines indicate when the CF, CD and MC mechanisms are active, respectively. (*b,d,f*) Final RI score depending on cell density at the final step of the simulation. Error bars represent s.d. for average RIs and densities. Red lines represent linear regressions (correlation coefficient: *r* = 0.308, *r* = 0.58 and *r* = 0.87 with *p* = 0.044, *p* < 0.001 and *p* < 0.001 for (*b*,*d*,*f*) respectively). The blue line in D represents a non-linear regression (a*x/*b* + *x*), while the horizontal dashed line represents the RI value under which no cell type of density higher than 125 is observed.

We demonstrate here that a realistic AB implementation of the CF can significantly increase the mosaic regularity compared to a random distribution (*p* < 0.001). Indeed, the average RI values rapidly increase from random levels (between 1.8 and 2) until reaching a value of 2.42 (± 0.09) at the end of the CF mechanism (figure 4*a*). However, such RI values are lower than the experimentally observed values (greater than 3), and so cannot be considered as solely responsible for the formation of regular mosaics. As shown in figure 4*b*, if CF is the only simulated mechanism, no correlation can be established between cell density and final RI values (correlation coefficient of 0.308, *p* = 0.044). Thus, mosaics of high cell density reach similar RI values as seen in mosaics of low cell density, as illustrated by the blue and orange lines in figure 4*a*.

#### 3.1.2. Cell death

**Figure 5.**
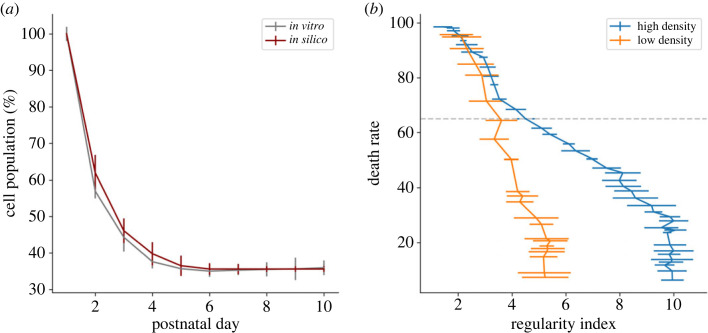
CD mechanism impact on RGC population. (*a*) RGC population measured *in vitro* (grey) and in simulations (dark red). *In vitro* population at day 1 is an estimation based on a final CD of 65%. Error bars represent s.d. (*b*) RI score depending on final CD rate in a simulation implementing only the CD mechanism, for selected RGC populations of high density (blue curve, initial density = 571 cells mm^−2^) and low density (orange curve, initial density = 114 cells mm^−2^). The horizontal dotted line indicates a death rate of 65%. Error bars represent s.d.

The CD is also able to significantly increase RI compared to a random distribution (*p* < 0.001), alone or in combination with the CF mechanism. The average RI value increases from random (around 1.8) to 3.31 (± 0.33) at the end of CD (figure 4*c*). This death rate amounts to around 65% when it reaches a steady state at the end of the simulation. These death rate dynamics are very similar to rates observed *in vitro* (figure 5*a*). Moreover, and unlike for the case of the CF mechanism, CD can generate mosaics of medium regularities (RI > 3).

Interestingly, and as shown by figure 5*b*, the death rate measured *in vitro* and selected for our simulations (grey horizontal dashed line) is not the one generating the highest regularity. The highest scores of RI are achieved for death rates between 5% and 30% of the RGC population, regardless of the initial density of the considered population. After 30% of CD, RI decreases until it reaches a random distribution once around 90% of cell death is achieved. This is observed under both high and low-density conditions and in simulations with CD alone or in combination with CF. Interestingly, and in contrast with the CF mechanism, we observe strong differences between populations of high and low initial densities. As shown in figure 5*b*, the high-density population can generate more regular mosaics than the population of low density, both for their maximum value (RI > 9 and RI > 5, respectively, when cell death is below 30%) and at 65% of cell death (RI = 4.49 ± 0.36 and RI = 3.61 ± 0.59, respectively). Therefore, a positive correlation between cell density and the final regularity is observed when the death rate is set to 65%. Mosaics of low density exhibit low RI values, while those of cell density higher than 65 cells mm^−2^ (vertical dashed line of figure 4*d*) exhibit a higher average RI score of 3.35 (horizontal dashed line of figure 4*d*).

While no differences are observed in RI scores between simulation of the CD mechanism and a combination of the CF and CD mechanisms if all mosaics are considered (3.31 ± 0.33 and 3.48 ± 0.44, respectively, *p* = 0.27), a positive impact on dense mosaics' regularity (for cell densities higher than 125 cells mm^−2^) is to be noted. Thereby, RI values in the case of CF and CD combination plateaus around 4.1 instead of 3.6 if CD is the only implemented mechanism. This result is in line with previous work reporting higher mosaic regularity using a combination of CF and CD compared to using CF or CD alone [11].

#### 3.1.3. Cell migration and combinations of mechanisms

A combination of all three mechanisms (CF, CD and CM) is also able to generate mosaics significantly more regular than random distributions (*p* < 0.0001).

A first RI increase corresponding to the effect of CF is observed (figure 4*e*). After the CD and CM mechanisms are triggered (first dashed line), they give rise to a significant second increase, until the RI value stagnates toward the end of CD (simulation day 4 to 5.5 depending on the cell type). Finally, a third RI increase is observed after CD completion (second dashed line) due to the CM mechanism, leading to an average RI score of 4.01 (± 0.75) at the end of the simulation. A significant difference is observed between simulations implementing CF and CD and simulations implementing CF, CD and CM (*p* < 0.0001). Unlike CF and CD mechanisms, this simulation condition can generate highly regular mosaics (RI > 5), thanks to tangential migration. Indeed, simulations implementing CM alone—even if this case does not account for the observed CD—generate mosaics of RI similar to simulations implementing all three mechanisms (RI = 4.12 ± 0.97, *p* = 0.43), as well as highly regular mosaic. Moreover, a strong correlation appears between cell density and RI values (linear correlation magnitude *r* = 0.87, *p* < 0.001) as shown by figure 4*f* when all mechanisms are implemented. Thereby, only RGC types exhibiting a cell density higher than 125 cells mm^−2^ can generate mosaics with a RI value higher than 5. Thus, as illustrated by the blue and orange lines in figure 4*e*, significant differences emerge between mosaics of high and low density. No significant differences are seen between simulations of CD and CM combination and simulations of CF, CD and CM combination. Final RI values for each mechanism alone and in combinations are illustrated in the electronic supplementary material, figure S2.

#### 3.1.4. Migration distance

**Figure 6.**
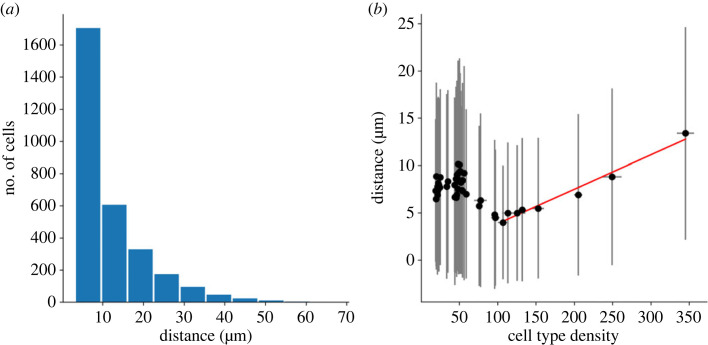
Migration distance measured in simulations implementing CF, CD and CM. (*a*) Migration distance distribution. (*b*) Relation between cell type density and migrating distance. The red line represents the correlation between migration distance and cell type density for densities higher than 100 cells mm^−2^ (correlation coefficient *r* = 0.92, *p* < 0.01).

When all three mechanisms are implemented, surviving cells migrate tangentially with an average distance of 8.72 µm (± 0.11, *n* = 8), which is in accordance with *in vivo* measurements reporting migration distance below 30 µm [31]. Important disparities in migration distance between cells are to be noted, as shown in figure 6*a*, with an average migration distance s.d. of 9.44 (± 0.18). No correlation between final RI and migration distance can be seen. Likewise, no correlation appears between final density and migration distance if the whole population is considered. However, if only populations with a final density higher than 100 cells mm^−2^ are considered, a correlation can be observed (correlation coefficient *r* = 0.92, *p* < 0.01; figure 6*b* red line). Hence, the denser the cell type the larger the distance cells migrate.

### 3.2. Starburst amacrine cell mosaic development

**Figure 7.**
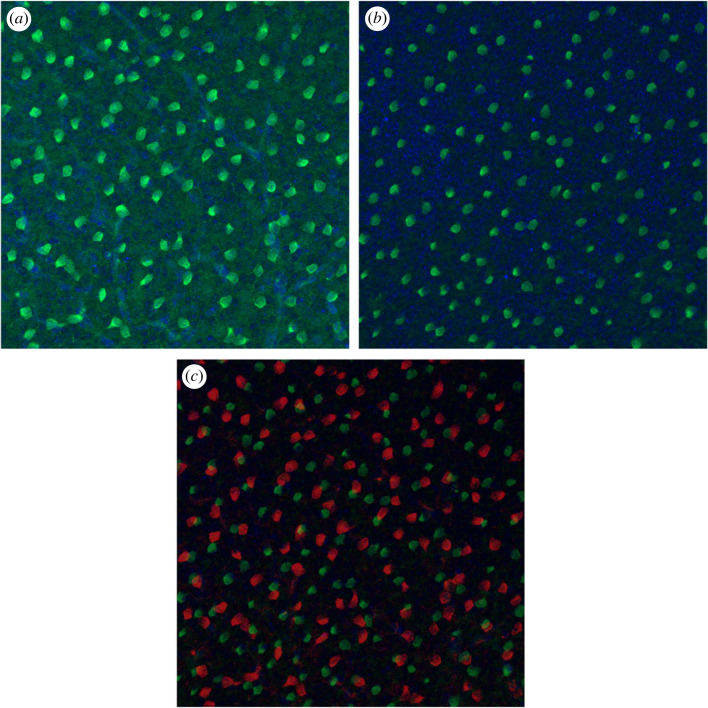
ChAT immunostaining on a P9 pup retina. (*a*) GCL level. (*b*) INL level. (*c*) Overlap of GCL (red) and INL (green) levels. GCL and INL level images are taken at the same *x*,*y* position, but at different depth focus. Regular SACs positioning can be observed in each cellular layer. Only few cells overlap between GCL and INL levels.

**Figure 8.**
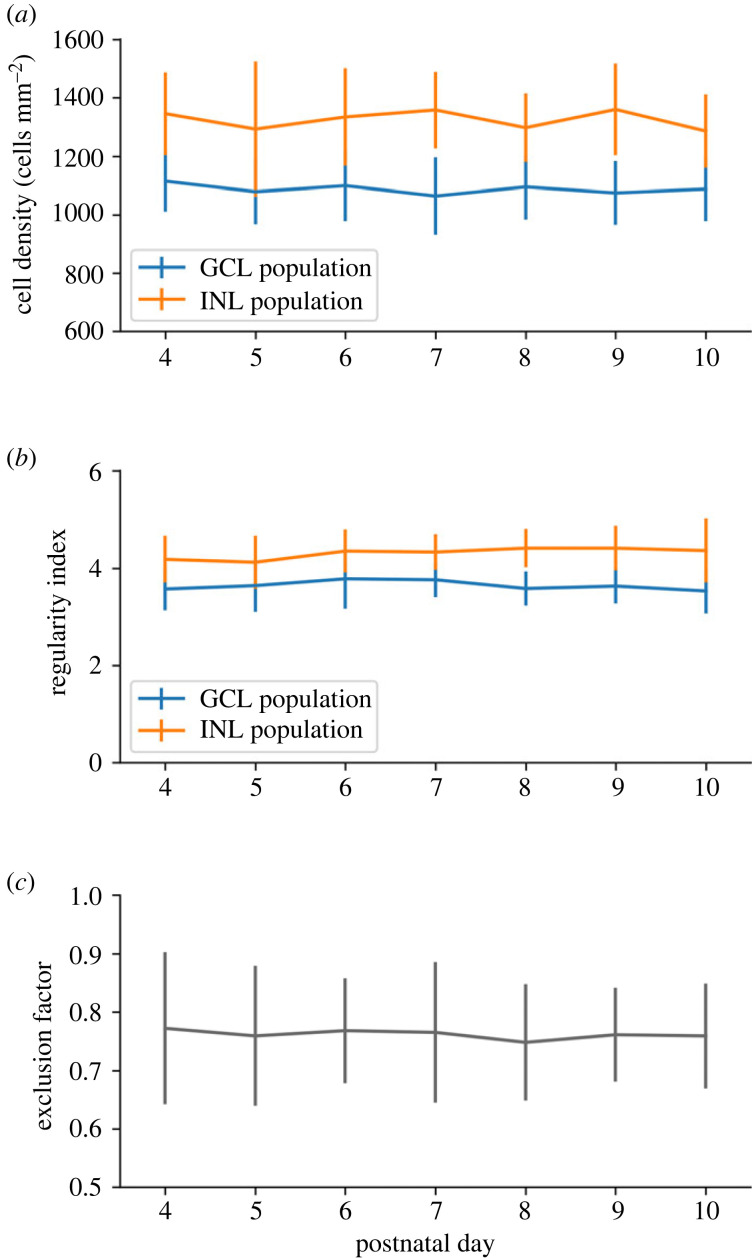
*In vitro* SAC population characteristics through development. (*a,b*) SACs in the CGL population are represented in blue, and the INL population is represented in orange. (*a*) Cell density over time. (*b*) RI over time. (*c*) GCL and INL SAC population exclusion. A score of 1 denotes two mosaics with a perfect exclusion and a score of 0 a total overlap of mosaics. Exclusion diameter of 32 µm. Error bars represent s.d. P4: *n* = 14; P5: *n* = 15; P6: *n* = 14; P7: *n* = 12; P8: *n* = 10; P9: *n* = 9; P10: *n* = 10. There are no significant differences between P4 and P10.

The SAC population is divided between two different cellular layers, the GCL and the INL, forming two separate populations (figure 7). Our *in vitro* results reveal no significant differences in the GCL and INL populations densities from P4 to P10 (*p* = 0.27 and *p* = 0.32, respectively; figure 8*a*). These two SAC populations exhibit regular pattern organization, and no significant difference over time of their RI is measured from P4 to P10, as shown by figure 8*b*. GCL and INL SAC mosaics are reported to be independent, in line with experimental data showing that SAC populations in the GCL and the INL only moderately overlap [1,2,37]. A measure of these populations’ exclusion has then been conducted, showing no significant difference from P4 to P10, as shown by figure 8*c*. This suggests that the INL and GCL SAC populations have already formed their mosaics from P4 (shortly after GCL and INL separation) and do not exhibit further significant CM once SACs have migrated to their respective cellular layer. For this reason, SAC mosaics formation is implemented before GCL/INL separation in our simulations.

Two different developmental conditions have been simulated, using either one common or two separated chemical substances for mosaics formation (one for GCL population, one for INL population). To assess if the GCL and INL mosaics overlap or exclude each other, we calculate an exclusion factor, as described in the Methods.

Interestingly, by using an identical concentration threshold triggering CM for SAC in the GCL and INL, the GCL SAC population exhibits less regular mosaics than the INL population at the end of the simulation. This is observed in both developmental conditions, using either one common or two separate chemical substances for mosaic formation (RI of 3.57 ± 0.12 and 4.11 ± 0.12, respectively, when one substance is used, RI of 3.36 ± 0.07 and 4.37 ± 0.15, respectively, when two substances are used, *n* = 8 for each group, *p* < 0.0001). This mosaic regularity disparity is in accordance with observations in mouse (figure 8*b*) where the INL population has been reported to be more regular than in the GCL population. In our simulations, this disparity can be explained by the cell density difference between these two layers. Indeed, and as previously demonstrated in our simulations, the denser a cell population is, the more regular its mosaic can be. Hence, our model provides a mechanistic explanation for this observed difference in RIs between the two SAC populations.

No significant differences are observed when the average closest cell of the other population is considered. This is the case when comparing the one cue and two cues simulation conditions, where the average closest cell distances are 242.3 µm (± 120.1) and 235.1 µm (± 104.8), respectively, (*p* = 0.15 with a Mann–Whitney *U*-test). Similarly, no differences are observed between the two simulation conditions and the average closest cell measured *in vitro* (average of 235.6 µm ± 99.1, *p* = 0.13 and *p* = 0.45, respectively, with Mann–Whitney *U*-tests).

However, we find that an important difference emerges between the two conditions concerning the exclusion factor of the two SACs populations: if one common developmental cue is used, GCL and INL mosaics exclude each other with a calculated exclusion factor of 0.71 (± 0.01, *n* = 8), similar to what has been measured *in vitro* (0.74 ± 0.09, *n* = 5; figure 7). This indicates that the GCL and INL populations' mosaics tend not to overlap, and so are not fully independent of each other. However, if two distinct developmental cues are used, the exclusion factor is lower, at 0.31 (± 0.1, *n* = 8), denoting independent mosaics that tend to overlap. In this second condition, the measured exclusion factor is significantly lower than the one observed in mouse (*p* < 0.0001, *n* = 8 and 5, respectively). Thus, only the first condition can reproduce the results observed *in vitro*.

## 4. Discussion

Using biological data from our *in vitro* experiments and from the literature, we built realistic simulations of retinal cell self-organization. The number of RGCs types incorporated in our simulations is based on evidence from the literature [3–5]. However, the literature is incomplete regarding precise information about characteristics of RGC types, especially for low-density RGCs. To define all the types of RGCs and the number of types to use in our simulations according to our current understanding of retinal cellular organization and function, we had to infer missing information from the literature. Sanes & Masland [5] speculated that known RGC types represent only about 60% of the total RGC population, corresponding to around 1740 cells mm^−2^ from the total 3000 cells mm^−2^ observed in the mouse retina. In addition, it is important to note that from these known RGC populations, only 12.4% are On type. As On, Off and On-Off are equally numerous (30% to 35% each), a great number of On cells still needs to be discovered in order to reach the theoretical percentage of On RGC in the total RGC population. Thus, we can hypothesize that: (i) Several high-density On types have not yet been discovered. (ii) There are more On types than Off or On-Off types.

The first hypothesis appears unlikely as RGCs are widely studied, especially with the emergence of large-scale and high-density MEA recordings [6], but also using morphological and molecular characterizations. Thus, it is unlikely that the existence of dense On RGC types (representing the majority of the On population, and so being the most common On type) has not been captured by at least one of these techniques. The second hypothesis appears to be supported by experimental evidence because mice, similarly to other nocturnal animals, have rod-dominated vision. Indeed, rods are known to project their dendrites and to establish synaptic connections only to On bipolar cells, that in turn establish synaptic connections to On RGCs. In order to extract as many features as possible from a visual scene using mainly rod vision, a great diversity of specialized RGCs can be justified. The hypothesis of a great diversity of low-density On types is also in agreement with Sanes & Masland [5], who speculate that around 30 low-density RGC types exist and are yet to be discovered. Baden *et al*. [3] also estimate the total number of RGC types to be over 40, supporting the hypothesis of numerous low-density RGC types. As it is still possible that On types of mid density has not been discovered, we chose to allow the possibility for this hypothesis in our simulations, in addition to adding multiple low-density On RGCs.

One major basis of our simulations is the presence of chemical cues supporting cellular self-organization mechanisms. Evidences of such chemical cues have been previously reported [1,38,39].

### 4.1. Retinal ganglion cell mosaic formation

The impact of the CF mechanism on RGC mosaics’ regularity is particularly difficult to study *in vitro* or *in vivo* as RGC progenitor cells do not express RGC type-specific markers cells will differentiate into. Despite experimental studies on RGC progenitors, no evidence has been found for RGC type-specific progenitors [40]. Hence, RGC types are probably not pre-determined early on and so are likely to depend on extrinsic factors, such as the presence of chemical cues [13]. Thereby, it allows for the contribution of a mechanism such as CF for RGC type differentiation and its potential implication in mosaic formation. One major conclusion from our simulations is that regular mosaics cannot be explained only through the CF mechanism. This suggests that RGC types are unlikely to be defined by cell body mosaics. They may instead be dictated by intrinsic factors (that remain to be discovered), functional determination (dictated by the input from other cells) or a combination of intrinsic factors interacting with extrinsic factors.

In our simulations, the CD mechanism can create regular mosaics (RI > 3.5) with a death rate of 65%. As this mechanism is based on a locally diffused chemical substance, homotypic cellular spacing (and therefore cell type initial density) has an important impact on the CD mechanism. For this reason, only populations with a high initial cell density exhibit regular mosaics. Importantly, our CD implementation matches measured RGC death dynamics during development, thus strengthening its plausibility. However, CD serves additional purposes in the retinal maturation process and is not only geared towards mosaic creation. Indeed, some cell types which do exhibit mosaic regularity do not undergo any significant levels of CD (such as horizontal cells or photoreceptors). In addition, as demonstrated here, the maximum positive impact of CD upon RI is reached at death rate lower than 30%, below the 60–65% death rate observed in mouse. This implies that even if CD can be involved in mosaic formation at early stages, cell death at levels above 30% is likely to be driven by other mechanisms and for other purposes than mosaic formation. For instance, CD could be implicated in refining retinal functional connectivity and activity. CD could also have evolutionary advantage with regard to generating an optimized neural architecture [41].

Finally, CM is the only mechanism able to explain the formation of highly regular mosaics (RI > 5). These results are in accordance with previous studies, demonstrating that mosaics can be formed from local interactions between cells [5]. Our simulations show that: (i) CM yields mosaics of higher regularity than CD. (ii) CD yields more regular mosaics than CF. (iii) A combination of the CF and CD mechanisms creates more regular mosaics than CF or CD alone, which is in accordance with the literature [5,13].

Similarly to the CD mechanism, the efficacy of the CM mechanism depends on cell density—as it is based on local interactions. The shorter homotypic cellular distances are, the more they can sense and repulse each other. Thereby, a strong correlation emerges between RGC type populations densities and the regularity of their mosaics. Therefore, we propose here that low-density RGC type mosaics exhibit on average significantly lower regularities than high-density RGC type mosaics. It would be very informative to experimentally verify this prediction. To this date, this question remains unanswered. This hypothesis is in accordance with recent studies showing that some low-density RGCs do not exhibit regular spacing [42].

Moreover, we question here the relevance of regular spacing as a criterion for a group of RGCs to form a RGC type. Indeed, if no low-density RGC types exhibit highly regular spacing as predicted here, this criterion does not discriminate RGC types. Retinal mosaics have been reported to enable uniform sampling of visual information [43,44]. Our finding could have implications in our understanding of how different features of a visual scene are extracted and processed by the retina. We can also interrogate how the existence of low-density RGC populations that are not organized in mosaics fits within the theory of efficiency coding, which has been proven to generate regular and anti-aligned RGC organizations [45,46].

We also show here that high mosaic regularity can be achieved with limited migration distance (8.72 µm ± 0.11, *n* = 8). This average migration distance is in accordance with *in vivo* measurements, reporting that RGCs and SACs tangential migration does not exceed 30 µm [31]. However, the average migration distance measured in our simulations is notably lower than the one experimentally measured at around 20 µm [27] and could be explained by the absence of retinal surface expansion implementation in our simulations. The CM mechanism implemented here is based only on local cues and short-distance interactions, and thereby follows the description of tangential dispersion in mouse, reported as a local, short-distance, phenomenon [28]. Our results are consistent with previous studies showing that a tangential cell dispersion does not appear to be directly related to the cell time of birth, but rather to its cell type [28].

**Figure 9.**
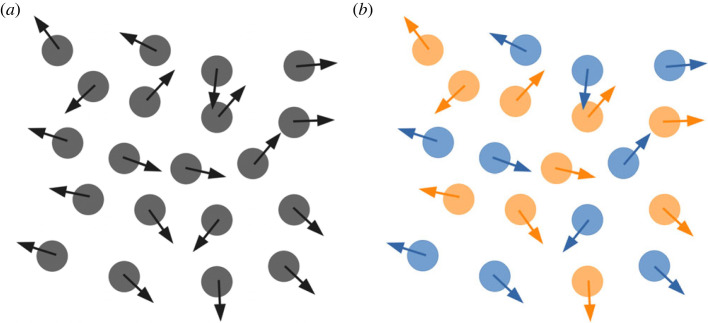
CM appearing as random if the whole population is considered homogeneously (*a*) or coherent (homotypic avoidance) if the population is sub-divided into two populations (*b*).

The CM and RI dynamics resulting from the CM mechanism are in agreement with the literature, where it is reported that RI increases mostly between P1 and P5, with the spacing between cells still increasing after that period, until P10 [4]. After reaching the correct cell layers, a slower and finer tangential positioning phase of RGC within the GCL has been reported [27,47]. Cellular movement during this period has been described as random but important for exact cellular positioning [47]. In accordance with our results and as stated by other studies [2], these highly varied movements are likely to be related to mosaic formation and refinement. Indeed, these movements appear random as the whole RGC population is considered, while it should be divided into types in order to meaningfully investigate RGCs lateral migration. If it were possible to examine each type independently, our model suggests that these movements, reported as random, would appear as coherent, as illustrated by figure 9.

### 4.2. Starburst amacrine cell mosaic formation

Besides RGCs, other retinal cell types are known to exhibit regular spacing, including photoreceptors, bipolar cells or SACs. While our simulation procedure can be applied to all cell types in the retina to study their spatial organization, we chose in this section to investigate SACs, which cell population is divided into the GCL and the INL. Both SAC layers form mosaics that are reported to be independent from each other [1,2]. Thus, there is only limited overlap between their populations. We found no RI variation from P3–P4, indicating that these two SACs populations have already created their mosaics by P3–P4, hence shortly after SACs migration into their respective cellular layer. Surprisingly, *in vitro* data did not show a decrease in cell density during the development while the retinal tissue is expanding, thus increasing in diameter, which theoretically should lead to a decrease in cellular density if no new cells are born. This entails the possibility for cell birth during that time, or perhaps CM. In addition, we observed that the calculated exclusion factor does not vary, also supporting this assumption. Moreover, the observed complementarity of GCL and INL mosaics perhaps indicates interactions between these two SAC populations during their cellular organization, before they migrate to their respective layer.

Here, we investigated this GCL–INL population interaction hypothesis further by building a simulation of SACs mosaics development. These simulations clearly show that our modelling procedure can successfully be applied to another cell population, with minimal parameter changes. We have been able to explain differences in GCL and INL mosaic regularities (the RI of the INL population being higher than that of the GCL population) by using only local interactions between SACs. This is the case if SACs constitute a unique population, or if GCL and INL populations are distinct (in other words, if one common or two distinct chemical cues are used). In the former case, this RI difference can be explained by the higher number of cells migrating to the INL compared to the GCL. Precisely, the percentage of a population characterized by a highly regular mosaic dictates the regularity of the resulting sub-population. Hence, the bigger the sub-population, the closer the obtained RI will be to the RI of the initial population, if cells constituting this sub-population are chosen randomly. In the latter case, this observed RI difference between the GCL and INL populations can be explained by the higher cell density of SACs in the INL. This higher cell density in the INL allows more interactions and homotypic repulsion and thus the emergence of a higher RI than for the cells located in the GCL.

However, and importantly, only the simulation condition using a common chemical cue for mosaic formation can explain the complementarity observed between the GCL and INL populations. Indeed, if the two mosaics are formed independently, they largely overlap without exhibiting the mutual exclusion observed *in vitro*. This suggests that the GCL and INL populations of SACs are not fully independent but anti-aligned. This result is in line with previous studies reporting that anti-alignment of mosaics with similar feature selectivity optimizes the encoding of visual scenes [45].

Our results predict that a shared guidance cue is responsible for mosaic formation of SACs in the GCL and INL. Locally diffused molecular guidance could be a possible cue candidate for mosaic formation. If this is the case, our prediction could be potentially experimentally verified by using knock-out experiments blocking either the secretion or the reception of this chemical guidance.

Our model does not aim at modelling and explaining mosaic formation at the whole retina's level. The focus of our model is on the local level and the explanation of how mosaics can form using only local cues and neighbouring cell–cell interactions. This work focuses on the early stage of mosaic formation and does not consider electrical activity, which is known to play a key role during retinal development [23,48,49]. Future work could be conducted using the same framework, investigating further retinal cellular organization by modelling axonal and dendritic development or by considering the impact of spontaneous electrical activity during retinal development. Notably, it has previously been demonstrated that spontaneous activity, such as retinal waves [50], is implicated in the establishment of retinal receptive fields [51]. Likewise, our model could be further extended with additional factors, including neurotransmitters and neuromodulators. In all cases, all mechanism added to the model should be informed by concrete experimental data, and the model should incorporate only mechanisms necessary to explain a given phenomenon—in order to limit its complexity and allow reliable interpretation of the results. Alongside experimental studies, computational modelling and the approach presented in this work, represent crucial tools to investigate the mechanisms taking place during retinal development.

## Data Availability

All the source code and data used to produce the results and analyses are available at https://github.com/JeandeMontigny/. The data are provided in the electronic supplementary material [52].
